# Nitrogen and Carbon Mineralization from Green and Senesced Leaf Litter Differ between Cycad and Angiosperm Trees

**DOI:** 10.3390/biology11121758

**Published:** 2022-12-03

**Authors:** Charles A. Paulino, Thomas E. Marler

**Affiliations:** 1 Western Pacific Tropical Research Center, College of Natural and Applied Sciences, University of Guam, Mangilao, GU 96923, USA; 2 Bagong Kaalaman Botanikal Institute, 15 Rizal Street, Barangay Malabañas, Angeles City 2009, Philippines

**Keywords:** *Cycas micronesica*, mineralization, *Morinda citrifolia*, soil priming

## Abstract

**Simple Summary:**

The addition of plant leaf litter to soils represents a critical component of the global carbon (C) and nitrogen (N) cycles. Tropical cyclones known at typhoons in the western Pacific islands transfer an enormous amount of green leaf litter from the forest canopy to the soil. We have shown on the island of Guam that this green litter releases carbon and nitrogen extremely rapidly compared with senesced leaf litter. Moreover, species such as Guam’s native cycad *Cycas micronesica* produce slowly decomposing litter, while other species such as Guam’s native angiosperm *Morinda citrifolia* produce rapidly decomposing litter. Soil priming is an important process in the global C cycle, and occurs when pre-existing organic matter releases more C as a result of new organic C additions. The green litter generated soil priming that was almost double that of the senesced litter. The release of N from the green litter into forms that are available to plants required only 14 d for the *M. citrifolia* litter but required more than 90 d for the *C. micronesica* litter. Scientists predict more frequent intense typhoons in the future, and we have shown one of the ecosystem-level processes that these typhoons will change.

**Abstract:**

Plant leaf litter decomposition is directly influenced by the identity of the source plants and the leaf age. Defoliation of forests by tropical cyclones (TC) transfers copious amounts of high-quality green leaf litter to soils. We used a soil amendment approach with the incubated buried bag method to compare carbon (C) and nitrogen (N) mineralization dynamics of green and senesced leaf litter from cycad *Cycas micronesica* and angiosperm *Morinda citrifolia* trees on the island of Guam. Soil priming increased the decomposition of pre-existing organic C, and were greater for green leaf litter additions than senesced leaf litter additions. Available N content increased by day 14 and remained elevated for the entire 117-d incubation for soils amended with green *M. citrifolia* litter. In contrast, available N content increased above those in control soils by day 90 and above those in soils amended with senesced litter by day 117 for green *C. micronesica* litter. The net N mineralization rate was higher than control soils by 120% for the senesced litter treatments and 420% for the green litter treatments. The results reveal a complex but predictable interplay between TC defoliation and litter quality as defined by tree identity. We have illuminated one means by which increased frequency of intense TCs due to climate change may alter the global C and N cycles.

## 1. Introduction

Plant litter inputs to the soil system directly influence the dynamics of organic matter decomposition. Litter decomposition speed is partly resolved by litter chemistry which determines litter quality [1]. Therefore, plant species identity is a key modulator of litter decomposition [2]. Additionally, terrestrial ecosystems in the western Pacific are frequently influenced by tropical cyclone (TC) activity [3]. These large-scale disturbances often defoliate forests, adding copious amounts of high quality green leaf litter to the soil system [4,5]. Altogether, the identity of both species and the frequency of TCs interact in the insular habitats of the western Pacific to determine litter decomposition dynamics.

One of the disciplines in research on global carbon (C) and nitrogen (N) cycling is the soil priming effect. The priming effect occurs when the decomposition of native soil organic C increases in response to the input of fresh organic matter to the soil system [6,7,8,9]. A dominant factor controlling the mineralization during the decomposition of bulk soil organic matter during the priming effect is the chemistry of the new litter additions [10]. Soil priming has been discussed as a mediator in global-scale models of the C cycle [11], hence priming research is needed to understand all unique ecosystems, such as Oceania.

Among the 25 sympatric taxa that were influenced by a TC in a coastal Guam forest, the cycad *Cycas micronesica* K.D. Hill exhibited leaf chemistry with low quality litter, whereas the angiosperm *Morinda citrifolia* L. exhibited leaf chemistry with high quality litter [12]. To our knowledge, there have been no studies comparing actual litter decomposition or mineralization of these two sympatric indigenous tree species or addressing how TC defoliation influences these processes. Based on the fact that *C. micronesica* litter chemical traits have predicted recalcitrance and *M. citrifolia* litter chemical traits have predicted lability, we have hypothesized that the decomposition differences between soil amended with green versus senesced litter would be contrasting for *C. micronesica* and *M. citrifolia* leaves. We predicted that there would be differences between the decomposition of green and senesced leaves, which would become evident more rapidly for *M. citrifolia* and would become evident more slowly for *C. micronesica*.

## 2. Materials and Methods

Leaf litter was collected in northeast Guam from trees growing in coastal karst substrates that were formed in slope alluvium, loess, and residuum overlying limestone (Clayey-skeletal, gibbsitic, nonacid, isohyperthermic Lithic Ustorthents) [13]. The litter was collected in January 2018 from a single area of occupancy, in which, *C. micronesica* and *M. citrifolia* trees coexisted. Fresh senesced litter was collected as yellow leaves rather than brown leaves to ensure no nutrient leaching of the tissues had occurred following senescence. The youngest fully expanded green leaves were also collected from the same trees which provided the senesced litter. The leaf material was cut into 2 cm sections and then stored in paper bags in a laboratory at the University of Guam.

The speed of C and N mineralization from these sources of leaf litter was determined using a soil amendment approach by employing the buried bag method [14]. Fresh soil was collected from the surface (top 15 cm) in a forested portion of the University of Guam campus on 1 September 2018. The soil was the same karst soil series as the litter collection site, and there were numerous tree species that had contributed litter. We restricted the soil collection to locations where there was no prior exposure to *C. micronesica* or *M. citrifolia* litter to ensure there were no complications from home-field advantage effects [15]. Large rocks were extracted by hand and small rocks were filtered out with a sieve. After which, the bulk soil was thoroughly homogenized.

The initial nutrient content in each of the four categories of leaf litter was determined from three separate samples removed from the bulk dried leaf litter. Each sample was milled to pass through a 20-mesh screen. Total N and total C were determined by dry combustion (LECO CN Analyzer, LECO Corporation, St. Joseph, MI, USA).

The living soil was separated into 15 3-L samples for incubation. The air-dried litter for each species and leaf age was milled to pass through a 20-mesh screen, which was then separated into 100-g samples. Each 100-g sample was added to one of three 3-L samples of soil and homogenized to create three soil amendment replications per species–leaf age combination. This approach utilized 12 of the soil samples, and the remaining 3 3-L soil samples served as controls with no added leaf litter. Thus, there were 5 soil treatments with 3 replications. Control soils were not amended. Green litter *M. citrifolia* soils contained 100 g of green *M. citrifolia* litter, green *C. micronesica* soils contained 100 g of green *C. micronesica* litter, senesced *M. citrifolia* soils contained 100 g of senesced *M. citrifolia* litter, and senesced *C. micronesica* soils contained 100 g of senesced *C. micronesica* litter.

We had previously determined that the water holding capacity of this soil was 41%. We added water to each 3-L replication to approximate a 50% water holding capacity to ensure adequate moisture and aeration. An initial soil sample of 100 mL was extracted from each of the 15 bags to serve as day = 0 in the incubation study. Each sample that was collected was enclosed in a plastic bag and frozen at −20 °C. The remaining soil for each replication was enclosed in a 3.78-L resealable plastic bag for incubations.

The 15 bags were buried in an 80 cm × 420 cm mesocosm constructed in an open air laboratory setting on 2 September 2018. The mesocosm was filled with horticultural perlite, in which each bag was buried, and a randomized complete block design was employed using three blocks. Each bag was opened and the soil was thoroughly mixed every two weeks to ensure that the decomposing organic matter was redistributed throughout the mineral soil and that the carbon dioxide did not accumulate in the bags. Fresh weights were used to determine if there was water loss, and water was added to maintain a 50% water holding capacity whenever needed. Additionally, 100-g samples were removed at 14, 30, 60, 90, and 117 days and frozen at −20 °C. Therefore, there were 6 sampling periods, including the initial sampling period.

For chemical analyses, the soils were thawed to ambient temperature, then N and C components of the soil samples were determined as previously described [16]. Total N and total C were determined by dry combustion [17], and organic C was determined by dichromate consumption using the modified Walkley–Black protocol [18]. Nitrate N and ammonium N were determined colorimetrically following 2*M* potassium chloride extraction [19]. Available N was determined as the sum of the nitrate and ammonium content. Both the percentage of total N comprised of available N and the percentage of total C comprised of organic C were calculated. In addition to these analyses, which were conducted for every sampling date, we also determined the content of other characteristics for the unamended control soils by combining all sampling dates for each of the three replications. Extractable macronutrients other than phosphorus (P) were quantified following digestion with diethylenetriaminepentaacetic acid [20]. Analysis was completed using inductively coupled plasma optical emission spectrometry (Spectro Genesis; SPECTRO Analytical Instruments, Kleve, Germany). Available P was determined using the Olsen method [21]. Soil reaction was determined from saturated paste (Accumet AB200 meter, Thermo Fisher Scientific, Singapore).

The influence of the five soil treatments (control, green *M. citrifolia*, green *C. micronesica*, senesced *M. citrifolia*, and senesced *C. micronesica*) over 117 d of incubation on the C and N variables were assessed using factorial analysis with repeated measures according to the variance observed in five species–leaf age combinations, six sampling dates, and three replications treated as blocks (PROC MIXED, SAS Institute, Cary, NC, USA). The incubation time was designated as the repeated measure. Five different covariance structures were evaluated for each response variable according to Akaike information criterion. Compound symmetry was determined to be the best covariance structure for total N, total C, and percent organic C. Heterogeneous compound symmetry was determined to be the best structure for ammonium N, nitrate N, organic C, and percent available N. Finally, unstructured covariance structure was found to be the best covariance structure for available N. Mean separation for significant factors was conducted by Tukey’s honest significant difference.

In addition to the response variables that were determined for every sampling date, we calculated the efficiency of priming by subtracting the final organic C content of each primed soil bag in a block from the finalorganic C content in the corresponding control soil bag from each block. This metric represented the amount of organic C in the control soils that was mineralized by the activities of microorganisms stimulated by priming with the fresh organic matter additions. We used a one-way analysis of the variance to determine the influence of the four species–leaf age soil amendments on the efficiency of priming. Mean separation was conducted using Tukey’s honest significant difference.

We also calculated net N mineralization for each of the five soil treatments by subtracting the initial available N concentration from the final available N concentration, then dividing by the 117-d incubation duration. This metric is the sum of the net ammonification and net nitrification, and represents the release of inorganic N from soil organic matter. We used a one-way analysis of the variance to determine the influence of the five soil treatments on net N mineralization. Mean separation was conducted using Tukey’s honest significant difference.

## 3. Results

The incubation soils without amendments exhibited characteristics that were similar to those reported for this series of karst soil. Total N was 3.4 ± 0.4 mg·g^−1^, total C was 72.3 ± 4.8 mg·g^−1^, Olsen phosphorus was 89.6 ± 8.8 µg·g^−1^, extractable potassium was 354.7 ± 34.6 µg·g^−1^, extractable calcium was 18.5 ± 1.6 mg·g^−1^, extractable magnesium was 1289 ± 78.2 µg·g^−1^, and soil pH was 7.5 ± 0.1.

The senesced *M. citrifolia* litter samples contained 13.6 ± 1.6 mg·g^−1^ N and the senesced *C. micronesica* litter samples contained 19.8 ± 2.8 mg·g^−1^ N. The senesced *M. citrifolia* litter samples contained 473.8 ± 11.9 mg·g^−1^ C and the senesced *C. micronesica* litter samples contained 509.8 ± 19.8 mg·g^−1^ C. The green *M. citrifolia* litter samples contained 25.1 ± 3.6 mg·g^−1^ N and the green *C. micronesica* litter samples contained 26.7 ± 3.8 mg·g^−1^ N. The green *M. citrifolia* litter samples contained 433.5 ± 14.9 mg·g^−1^ C and the green *C. micronesica* litter samples contained 496.7 ± 21.8 mg·g^−1^ C.

### 3.1. Carbon Changes with Duration of Incubation

The total C, organic C, and the percentage of total C comprised by organic C were all influenced by the type of soil treatment (Table 1). Additionally, the organic C and percentage of C that was organic were also influenced by the time of sampling. The interaction of these two main factors (S × T) was not significant for any of the C response variables, therefore the changes related to incubation time were similar for each of the soil treatments.

**Figure 1:**
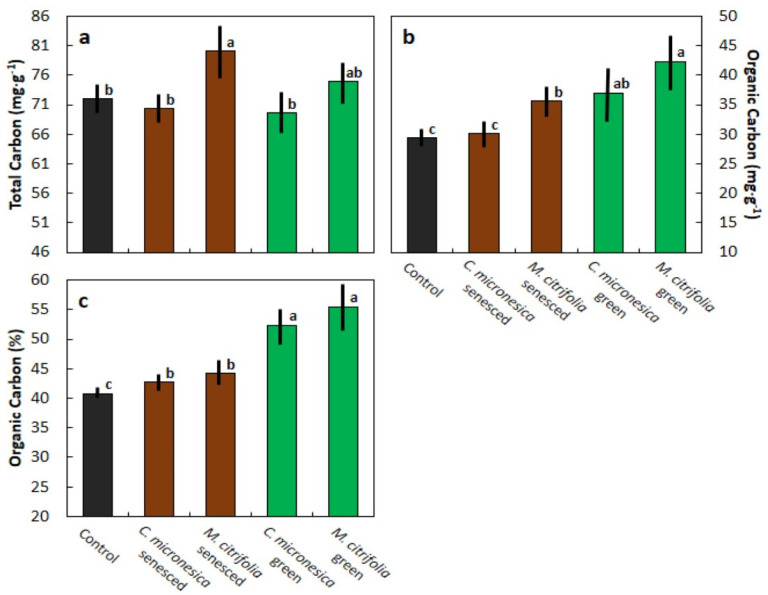
The influence of soil amendments with *Cycas micronesica* or *Morinda citrifolia* leaf litter on soil carbon traits. (**a**) Total C; (**b**) organic C; (**c**) percentage of total C comprised of organic C. Bars with same letter are not significantly different according to Tukey’s HSD. Mean ± standard error, *n* = 18.

The total C of the primed soil treatments are separated into two overlapping groups (Figure 1a). The soils primed with either the green or senesced leaf age categories of *M. citrifolia* exhibited the greatest total C throughout the 117-d incubation period. Soils primed with either of the two leaf age categories of *C. micronesica* exhibited a total C that was not different from that in the control soils. The organic C content of the primed soil treatments are separated into three groups (Figure 1b). The soils primed with green *M. citrifolia* leaf litter exhibited the greatest organic C content, followed by the soils primed with senesced *M. citrifolia* or green *C. micronesica* leaf litter. Organic C content of soils primed with senesced *C. micronesica* leaf litter did not differ from that in the control soils. The percentage of total C that was comprised of organic C can be separated into three groups (Figure 1c). The two green leaf litter priming treatments exhibited the greatest percentages, the two senesced leaf litter priming treatments exhibited intermediate percentages, and the control soils contained the least percentages of organic C.

**Figure 2:**
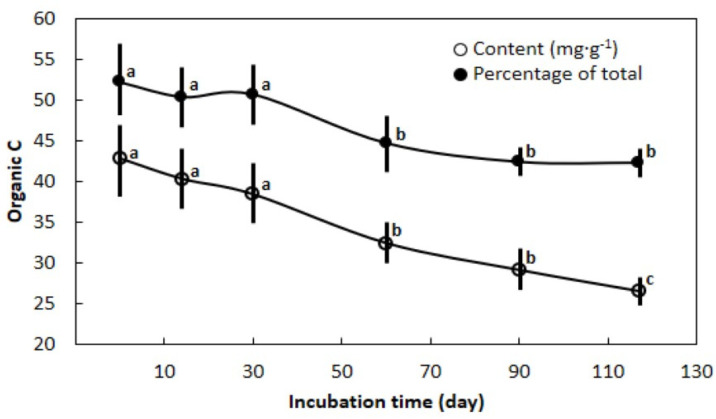
The influence of soil amendment with *Cycas micronesica* and *Morinda citrifolia* leaf litter on changes in carbon status over a 117-d incubation period. Means with the same letter are not statistically different according to Tukey’s HSD. Mean ± standard error, *n* = 18.

The total C content in the primed soils was not influenced by the main factor of time and exhibited an overall mean of 73.5 mg·g^−1^. In contrast, organic C content in the primed soils declined 38% from day 0 to day 117 (Figure 2). Similarly, the percentage of total C comprised of organic C declined as the incubation duration progressed. The initial organic C percentage was 1.23-fold greater than the final organic C percentage.

### 3.2. Nitrogen Changes with Duration of Incubation

Total N content in the incubation soils was not influenced by the main effect of soil treatment or incubation time (Table 2). In contrast, the interaction of the treatment–incubation time (S × T) exerted a significant influence on the total N content. Ammonium N, nitrate N, available N, and the percentage of total N comprised of the available N were influenced by the main effect of soil treatment, the main effect of incubation time, and the interaction of the treatment–incubation time (Table 2).

**Figure 3:**
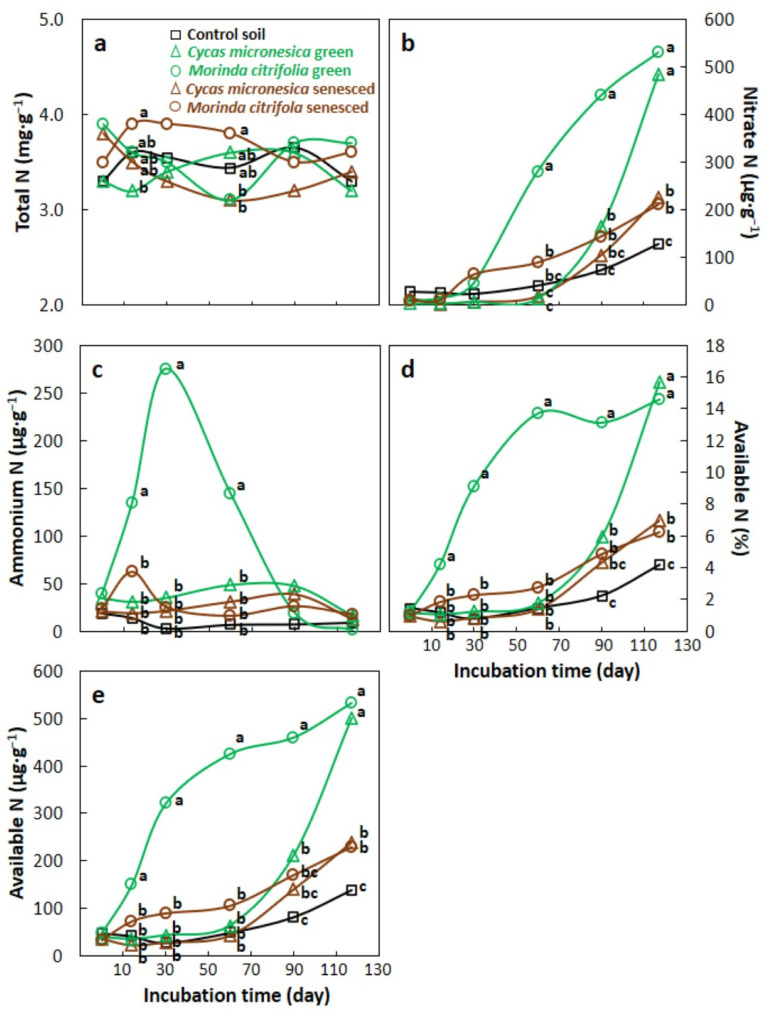
The influence of soil priming with *Cycas micronesica* or *Morinda citrifolia* leaf litter on changes in the nitrogen status over a 117-d incubation period. (**a**) Total N; (**b**) nitrate N; (**c**) ammonium N; (**d**) the percentage of total N comprised of available N; (**e**) available N. Means with same letter within each sampling date are not statistically different according to Tukey’s HSD. Dates with no letters indicate no differences among the soil treatment means.

Total N was limited in the incubation substrate and changed minimally throughout the 117-d incubation period. The means separated into two overlapping significantly different groups on days 14 and 60, but were not different among the other sampling dates (Figure 3a). Nitrate N did not differ among the soil treatments until day 60 when the nitrate N of green *M. citrifolia* soils was triple that of the other soil treatments (Figure 3b). This pattern persisted until day 90, but by day 117 the soils primed with green *C. micronesica* leaf litter exhibited nitrate N that was not different from that in the soils primed with green *M. citrifolia*, and the soils primed with the senesced litter of both species exhibited nitrate N that was intermediate. All four primed soil treatments contained more nitrate N than the control soil treatment on day 117. Ammonium N increased rapidly in the soils primed with green *M. citrifolia* leaf litter and reached a peak on day 30 (Figure 3c). By day 60 these *M. citrifolia* soils still contained ammonium N that was more than triple that of the ammonium N in the other four soil treatments. The five soil treatments did not differ in ammonium N content on days 90 and 117. The percentage of total N comprised of available N was greater in the soils primed with green *M. citrifolia* leaf litter than for the other soil treatments on day 14 through day 90 (Figure 3d). On day 117, the means of this metric separated into three groups, with the soils primed with green litter from both species exhibiting the highest values, the control soils exhibiting the smallest value, and the soils primed with senesced litter from both species exhibiting intermediate values. The trends in available N content (Figure 3e) were similar to those of the percentage of available N (Figure 3d). The early surge in ammonium N content in the soils primed with green *M. citrifolia* leaf litter combined with the later surge in nitrate N content in these soils to generate elevated available N from day 14 to day 90. The rapid increase in nitrate N content from day 90 to day 117 in the soils primed with green *C. micronesica* leaf litter caused the available N content to converge for the two green leaf litter soil treatments.

### 3.3. Soil Priming Effect and Net N Mineralization

**Figure 4:**
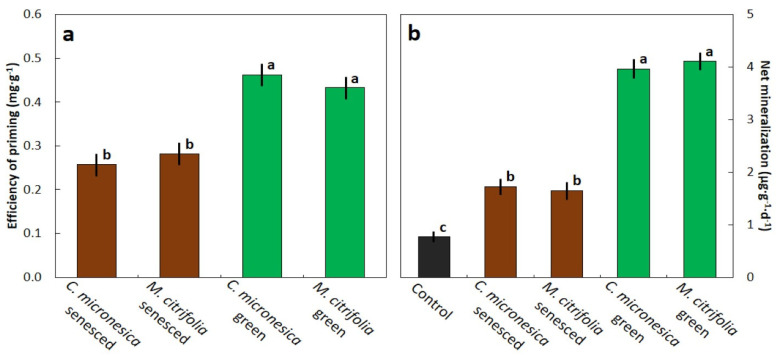
The influence of soil priming with *Cycas micronesica* or *Morinda citrifolia* leaf litter on changes in the nitrogen status during a 117-d incubation period. (**a**) efficiency of priming; (**b**) net mineralization. Bars with same letter are not statistically different according to Tukey’s HSD. Mean ± SE, *n* = 3.

The efficiency of the priming effect differed among the four primed soil treatments (*f*_3,8_ = 115.9; *p* < 0.001). Soils primed with the green leaf litter of both species contained about 0.45 mg·g^−1^ less organic C than the control soils at the end of the incubation period (Figure 4a). Soils primed with the senesced leaf litter of both species were not as efficient, and contained about 0.25 mg·g^−1^ less organic C than the control soils. These values suggest that the amount of organic C in the control soils was mineralized as a result of the priming effect.

Net mineralization differed among the five soil treatments (*f*_4,10_ = 690.9; *p* < 0.001). Soils primed with the senesced leaf litter of both species exhibited a daily production of available N that was 2.2-fold greater than that in the control soils (Figure 4b). Soils primed with the green leaf litter of both species exhibited a daily production of available N that was 5.1-fold greater than that in the control soils.

## 4. Discussion

On a global scale, soils contain more C than vegetation and the atmosphere [22,23], and plant litter inputs and decomposition represent a critical part of the role of soils in the C cycle [24]. Therefore, understanding the soil processes which promote soil C sequestration [25] and research into how tree species influence organic C in soils [26] are needed to mitigate climate change. We have shown that TC defoliation in the Mariana Islands of Guam is a consequential factor in understanding the C cycle of the region. As predicted by green and senesced leaf litter chemistry [12], mineralization of C in soils amended with green leaf litter was more rapid than in those soils amended with senesced leaf litter. Moreover, the priming effect of green leaf litter amendment was almost double that of the senesced leaf litter, representing the amount of native C that would have remained in soil organic matter if the litter additions had not occurred. We have also shown that the influence of species on litter quality exerted a direct influence on the early dynamics of N mineralization processes. The rate of increase in N mineralization for green litter was more rapid for the labile *M. citrifolia* litter than for the recalcitrant *C. micronesica* litter. In other words, the higher rate of litter decomposition of senesced *M. citrifolia* leaf litter was magnified by introducing green *M. citrifolia* litter to the soil system.

The components of available N were most contrasting during the early stages of decomposition. Net ammonium content increased almost 600% in only 30 d for soils amended with green *M. citrifolia* litter. In contrast, net ammonification of the soils amended with green *C. micronesica* litter kept pace with the nitrifying microbes, such that the ammonium content was never higher than that in the senesced leaf litter treatments. Similarly, net nitrification was evident earlier in the incubation for the soils primed with green *M. citrifolia* litter, which caused nitrate N to become elevated by day 30. This contrasted sharply with the green *C. micronesica* litter soils, which did not exhibit increased nitrate N until day 117.

The biodiversity of sympatric plant species enables heterogeneous litter components, and an additive effect of litter mixing has been heavily studied [27,28,29]. Our results illuminate how mixing different types of litter may also directly influence the availability of nutrients through the contrasting influence of initial mineralization processes. More research is needed to determine the influence of mixing these two litter sources. This is because the rapid accumulation of microbes responsible for ammonification caused by the green *M. citrifolia* litter additions may increase ammonification of the green *C. micronesica* litter and advance the mineralization process for the cycad litter. Our results provide an example of how spatiotemporal population levels of detritovore and decomposer soil microbe taxa may be dramatically influenced by litter identity.

Our methods did not quantify small organic molecules in the soils that may directly influence plant nutrition. For example, peptides and amino acids, which are sources of soil N, may be directly absorbed by some plant roots [30,31], mycorrhizal fungi [32,33,34,35], and non-mycorrhizal fungi and are known to partner with plant roots [36]. Leaf N resorption processes during senescence are primarily enabled by the hydrolysis of soluble proteins [37,38]. Therefore, a greater proportion of green leaf litter N may be contained in large organic N compounds because the hydrolysis of N-containing compounds to enable resorption of N is negated by TC defoliation prior to senescence. This larger pool of N macromolecules in green leaves than in senesced leaves may supply the soils with more peptides and amino acids, which may be absorbed by plant roots. These issues deserve further study.

The buried bag method was useful for understanding some of the dynamics of litter additions to this living soil in Guam. However, these methods disallowed the plant root uptake of newly available inorganic N and they disallowed the leaching losses of nitrate N. In situ mineralization studies need to be conducted to determine if the green and senesced leaf litter from these two indigenous tree species exert direct influences on N loss from the soils in a real ecological setting. The results predicted that the leaching losses from soils amended with green *M. citrifolia* litter may be less than the other soil treatments because much of the newly available inorganic N remained as ammonium N and was not readily nitrified. In contrast, the initial increases of inorganic N were primarily nitrate N for the green *C. micronesica* litter treatment and the senesced litter treatments for both species, and hence, nitrate is highly susceptible to leaching loss. These differences indicated that the nitrogen use efficiency may be greater for soils amended with green *M. citrifolia* litter because much of the available N was present in the root zone in a form that was less susceptible to leaching loss prior to plant root uptake.

The two model species we selected for this pilot study also illuminated an example of the cascading influences on ecosystem function caused by invasive species. Guam’s *C. micronesica* population has been decimated since the armored scale *Aulacaspis yasumatsui* Takagi invaded Guam in 2003 [39]. The *C. micronesica* population is the only native plant host for this invasive species. Moreover, herbivory of *C. micronesica* stems by feral pigs (*Sus scrofa* L.) increased after plant health was compromised by the armored scale herbivory [40]. In contrast, Guam’s *M. citrifolia* population has benefitted from endozoochory by feral pigs [41]. Guam forest inventories conducted in 2002 [42] and 2013 [43] corroborated these species-specific responses by documenting a 59% decrease and 26% increase in *C. micronesica* and *M. citrifolia* large trees, respectively, with a breast-height diameter greater than 12.7 cm. These invasive consumer species may indirectly exert profound influences on C and N cycling in Guam’s forests by continuing to reduce the recalcitrant *C. micronesica* litter inputs and increase the labile *M. citrifolia* litter inputs relative to other sympatric tree species.

## 5. Conclusions

Slowly decomposing leaf litter from trees such as *C. micronesica* provide an ecosystem service by enabling soil organic matter accumulation. Our results show that the green litter that is generated by TCs may mute the efficacy of that ecosystem service by increasing litter decomposition speed and increasing the C mineralization of pre-existing organic matter through the priming effect. The speed of N mineralization was influenced more by species than the speed of C mineralization, indicating localized shifts in C/N stoichiometry during the weeks following TCs. Our results revealed a complex but predictable interaction of TC defoliation and litter quality as defined by tree identity. We have illuminated one process by which increased frequency of intense TCs due to climate change may alter the C and N cycles in western Pacific islands.

## Figures and Tables

**Table 1:** Results from 5 soil treatment × 6 incubation time factorial ANOVA for soil carbon (C) variables. *n* = 3.

Response Variable	Soil Treatment *f*_4,58_	Soil Treatment *p*	Time *f*_5,58_	Time *p*	S × T *f*_20,58_	S × T *p*
Total C	3.5	0.049	1.8	0.122	1.2	0.332
Organic C	13.7	<0.001	12.4	<0.001	1.1	0.395
Percent organic C	7.8	0.007	5.1	<0.001	1.6	0.092

**Table 2:** Results from 5 soil treatment × 6 incubation time factorial ANOVA for soil nitrogen (N) variables. *n* = 3.

Response Variable	Soil Treatment *f*_4,58_	Soil Treatment *p*	Time *f*_5,58_	Time *p*	S × T *f*_20,58_	S × T *p*
Total N	3.7	0.054	1.6	0.169	2.3	0.010
Ammonium N	109.3	<0.001	56.6	<0.001	51.1	<0.001
Nitrate N	151.5	<0.001	203.4	<0.001	22.3	<0.001
Available N	269.6	<0.001	202.0	<0.001	105.7	<0.001
Percent available N	68.1	<0.001	111.0	<0.001	13.0	<0.001

## Data Availability

Data available upon request.
